# High Mobility and Low Use of Malaria Preventive Measures among the Jarai Male Youth along the Cambodia–Vietnam Border

**DOI:** 10.4269/ajtmh.15-0259

**Published:** 2015-10-07

**Authors:** Charlotte Gryseels, Koen Peeters Grietens, Susan Dierickx, Xa Nguyen Xuan, Sambunny Uk, Melanie Bannister-Tyrrell, Suzan Trienekens, Joan Muela Ribera, Susanna Hausmann-Muela, René Gerrets, Umberto D'Alessandro, Tho Sochantha, Marc Coosemans, Annette Erhart

**Affiliations:** Institute of Tropical Medicine, Antwerp, Belgium; Amsterdam Institute of Social Science Research, Amsterdam, The Netherlands; Partners for Applied Social Sciences (PASS) International, Tessenderlo, Belgium; School of International Health Development, Nagasaki University, Nagasaki, Japan; National Institute for Malariology, Parasitology and Entomology, Hanoi, Vietnam; National Center for Parasitology, Entomology and Malaria Control, Phnom Penh, Cambodia; Medical Research Council Unit, Fajara, The Gambia; London School of Tropical Medicine and Hygiene, London, United Kingdom; University of Antwerp, Antwerp, Belgium

## Abstract

Malaria control along the Vietnam–Cambodia border presents a challenge for both countries' malaria elimination targets as the region is forested, inhabited by ethnic minority populations, and potentially characterized by early and outdoor malaria transmission. A mixed methods study assessed the vulnerability to malaria among the Jarai population living on both sides of the border in the provinces of Ratanakiri (Cambodia) and Gia Lai (Vietnam). A qualitative study generated preliminary hypotheses that were quantified in two surveys, one targeting youth (*N* = 498) and the other household leaders (*N* = 449). Jarai male youth, especially in Cambodia, had lower uptake of preventive measures (57.4%) and more often stayed overnight in the deep forest (35.8%) compared with the female youth and the adult population. Among male youth, a high-risk subgroup was identified that regularly slept at friends' homes or outdoors, who had fewer bed nets (32.5%) that were torn more often (77.8%). The vulnerability of Jarai youth to malaria could be attributed to the transitional character of youth itself, implying less fixed sleeping arrangements in nonpermanent spaces or non-bed sites. Additional tools such as long-lasting hammock nets could be suitable as they are in line with current practices.

## Introduction

There is increasing evidence that minority groups and settings are key to malaria elimination as malaria risk is unequally distributed among populations^1^–^6^ leading to notable heterogeneity of burden within small areas. The factors contributing to the micro-epidemiology of malaria, including the substantial variation in malaria risk between neighboring villages^7^,^8^ or even households^9^ are still not fully understood but include variation in distance from the nearest mosquito breeding site, wind direction, and human genetic factors.^10^,^11^ In addition, the potential underlying human behavioral factors have seldom been investigated and include local preferences for housing construction,^12^–^15^ uptake of preventive measures,^2^–^4^ human mobility,^16^,^17^ and the presence of specific socially vulnerable or marginalized groups.^18^ For countries with decreased transmission moving toward elimination, these pockets of transmission (or “hotspots”) and their multifactorial determinants have become increasingly important to understand and tackle.^9^,^10^,^19^,^20^ Standard approaches such as indoor residual spraying (IRS) and long-lasting insecticidal nets (LLINs) are less likely to be as effective in some of these specific settings because of both human (mobility, housing structures, and low uptake of preventive measures) and mosquito behavior (early and outdoor transmission).^8^,^18^,^21^,^22^

In southeast Asia, despite improved malaria control, a major challenge for malaria elimination is the high mobility of populations in specific settings such as border regions, which are often inhabited by impoverished ethnic minorities largely dependent on the forest for subsistence, as is the case in the Vietnamese and Cambodian highlands.^16^,^23^–^29^ Moreover, the presence of *Anopheles dirus*, the main vector in these forested areas, challenges the effectiveness of standard control measures such as LLINs and IRS because of its outdoor and early biting behavior.^2^,^16^,^30^–^39^ In addition, the presence of an international border creates an artificial situation where commercial opportunities and kinship relations foster uncontrolled cross-border population movements that may influence malaria transmission. Considering that Cambodia and Vietnam are engaged in malaria elimination in the face of mounting artemisinin resistance, identifying potential risk groups for local transmission and those that may additionally carry these parasites across borders is all the more relevant.

This mixed methods study aimed at understanding sociocultural factors related to malaria infection along the Vietnamese–Cambodian border. The research was part of a bi-national Border Malaria Project launched in 2008 investigating cross-border malaria transmission between Ratanakiri (Cambodia) and Gia Lai (Vietnam) provinces.

## Methods

### Study site and population.

The study was carried out along the Vietnam–Cambodia border, namely in the villages of Phi, Old Lom, and New Lom in the district of Oyadao in Ratanakiri Province, and in the villages of Bi, Nu, and Son belonging to Duc Co District in Gia Lai Province. The selection of the study sites was based on their proximity to the border, the presence of ethnic minorities potentially crossing the border and engaging in forest activities, and malaria endemicity. All study villages on both sides of the border belonged to Jarai territory, and as such all participants were of Jarai ethnicity. The Jarai are traditionally dedicated to slash-and-burn farming in the forest. However, the region is undergoing rapid socioeconomic changes because of the newly constructed road connecting Pleiku City (Gia Lai) to Banlung City (Ratanakiri), and the designation of Ratanakiri and Gia Lai provinces as a special economic border zone by the Cambodia–Laos–Vietnam Development Triangle Master Plan.^38^

Malaria transmission in the study area is perennial, with peaks in June–July and October–November. The main malaria vector is *An*. *dirus* s.s., a sylvatic and highly efficient vector.^8^,^36^ Malaria prevalence by light microscopy was estimated at about 3% in the Vietnamese villages and about 6% in the Cambodian villages, of which the majority was infected with *Plasmodium falciparum* (Annette Erhart, personal communication). In Vietnam, at the time of the study, conventional insecticide-treated nets (ITNs) were provided free of charge by the National Malaria Control Program (NMCP), while in Cambodia, the NMCP provided LLINs free of charge. In both settings, village malaria workers from the community provide rapid diagnostic tests free of charge. In Ratanakiri, they also provide antimalarial treatment free of charge after a positive test, while in Gia Lai positive cases were referred to the commune health center.

### Research design.

The research consisted of a mixed method sequential design (in standard annotation [qual→quan])^40^ in which quantitative survey data were collected to confirm and quantify results from prior qualitative ethnographic research. During a first strand, ethnographic data were collected in local communities to acquire an in-depth understanding of malaria exposure in the study setting and population. The consecutive quantitative strand included two separate surveys, one targeting the Jarai youth (hereafter the “Youth Survey”) and the other, adult (married) household leaders (hereafter “Household Survey”). The objective of these surveys was to evaluate the following two hypotheses based on preliminary qualitative data: 1) Jarai youth use preventive measures less often than other age groups and 2) male Jarai youth constitutes a potentially high-risk group for malaria infection because of their sleeping patterns, including spending nights outside their families' homes while using little to no preventive measures.

### Data collection and sampling.

#### Qualitative data collection.

Ethnography was carried out in all six study villages over a total period of 5 months between 2008 and 2010, including participant observation and in-depth interviewing and was concomitantly complemented with additional interviews in Pak Touch village in the Oyadao health district and in the commercial center of Oyadao District, as these were located along the road to the study villages and were the central places for local commercial activities. A total of 257 interviews were recorded and transcribed, focusing on aspects such as the local housing system, mobility patterns, risk factors for malaria, and the use of preventive measures, and more specifically, on the Jarai youth social context. Participant observation consisted of daily life observations and reiterated informal conversations during field stays in the study villages. This technique was used to detect unforeseen variables and to contrast stated opinions with actual behavior, constituting a respondent independent data collection tool.

#### Sampling.

Following the principle of gradual selection, informants were theoretically selected (in accordance with emerging results/theory) and categorized in relation to relevant criteria (such as gender, age, locality, forest activities, previous malaria experience, and use of preventive measures). To increase confidentiality with respondents and consequent reliability of the data, “snowball” sampling techniques—sampling using participants to identify additional respondents—were used.

#### Analysis.

Qualitative data analysis was an iterative process performed concurrently with data collection. Preliminary data were intermittently analyzed in the field, and preliminary results were then translated into the question guides for follow-up interviews. Continuous validity checks were used to confirm or refute initial results until saturation was reached and the data could be theoretically supported. Analytic induction involved the iterative testing of theoretical ideas, which was used to refine and categorize themes grounded in the data while emerging themes were evaluated in dialogue with existing social science theory. This resulted in an analytical framework that was then systematically applied in the data analysis. Data were entered, managed, and analyzed in NVivo 8 Qualitative Data Analysis software (QSR International Pty Ltd., Cardigan, United Kingdom).

### Quantitative data.

#### Data collection.

In Cambodia, the Youth Survey was carried out in 2010 with all youth identified first based on the 2008 population census and consequently through systematic house-by-house visits in all the study villages. In Vietnam, the Youth Survey was carried out in 2010 with all youth identified using the 2008 population census. Youth was defined as any unmarried individual, male or female, aged 10–25 years.

For the Household Survey in Cambodia, all households in the villages that were listed in the population census were visited, after which the list was updated with all non-registered households. In Vietnam, the households were selected from the population census. During this survey, all household leaders—defined as the adult married men, constituting the family head—were interviewed.

The same closed-ended questionnaire on the use of preventive measures and types of mobility was administered face-to-face to both Youth and Household leaders, and the type and status of bed nets that respondents were using was directly observed by the interviewers.

#### Sampling.

In Cambodia, all 246 households living in the three study villages were included in the Household Survey in 2011 (four households were revisited three times, but household members were not found and therefore excluded). The Household Survey in Vietnam did not include all households because of logistical constraints; therefore a random sample of 203 (70%) households among the total 291 living in the three study villages was selected from the census.

#### Analysis.

Quantitative survey data were entered and cleaned in Epi Info 6.04 for Vietnam and in MS Excel for Cambodia. Data from both surveys were merged in a single database and analyzed in Stata 13 (StataCorp LP, College Station, TX) using the “svy” command to allow for the clustering effect at village level. Descriptive statistics were computed to summarize the main variables from both surveys and presented separately for Vietnam and Cambodia. Five different subgroups were compared: 1) household leaders, 2) male adolescents, 3) female adolescents, 4) male adolescents sleeping outside the parental home, and 5) male adolescents not sleeping outside the parental home. Differences between household leaders and adolescents, between male and female adolescents, and between males sleeping or not sleeping outside their parents' house were tested for each country separately using svy-χ^2^ test.

#### Case definitions.

The social category of “youth” was defined as the transitional phase between a parent-dependent, nonreproductive childhood and full integration into adult society through marriage, aligning with local understandings of youth and adulthood. Household leaders were defined as the married men constituting the head of the family. Qualitative data initially showed that adolescents from the age of 10 years and upward start undergoing a process of gradual independence from their parents, which manifest in many aspects including the tendency to sleep outside their parental houses.

Bed net protection was defined in three categories (optimal, partial, and no protection) by different combinations of the following four variables: 1) having a bed net (yes/no); 2) bed net use (always, sometimes, and never); 3) net type (LLINs, ITNs, or non-treated nets [NTNs]); and 4) net status (intact, torn). Individuals were defined as having “Optimal Protection” if they met all of the following three conditions: 1) had a net, 2) always used the net, and 3) used an LLIN or an ITN that was intact. The “No Protection” category included all participants who 1) either did not have a net or never used a net; 2) had a net; and, at the same time, 3) always or sometimes used a torn NTN. All other individuals fell under the “Partial Protection” category.

### Ethical considerations.

The study protocol was approved by the ethical committees of the Institute of Tropical Medicine and the University of Antwerp, Antwerp, Belgium, the Ministry of Health, Cambodia, and the National Institute of Malariology, Parasitology and Entomology, Hanoi, Vietnam. The interviewers followed the Code of Ethics of the American Anthropological Association (AAA).^41^ All interviewees were informed before the start of the interview about project goals, the topic and type of questions, the intended use of results for scientific publications as well as their right to reject being interviewed, to interrupt the conversation at any time, and to withdraw any given information during or after the interview. Anonymity was guaranteed and confidentiality of interviewees assured by assigning a unique code number to each informant. As proposed by the AAA, the interviewers sought oral rather than written consent from all interviewees since the act of signing one's name when providing certain information can be considered a potential reason for mistrust and may stigmatize illiterate informants.

## Results

### Survey participants.

In Cambodia, 300 Jarai youth participated in the Youth Survey, with slightly more males (*N* = 162) than females (*N* = 138), and a median age of 15 years (interquartile range [IQR] = 13–17). All 246 male household leaders were included in the Household Survey. In Vietnam, 198 youth participated in the Youth Survey, with more males (*N* = 111) than females (*N* = 87), and a median age of 14 years (IQR = 12–16). All 203 household leaders from the 70% sampled households participated in the Household Survey.

### Jarai housing structures and mobility.

On the basis of the ethnographic study, Jarai families combine sleeping in village homes (traditionally longhouses or “sang”) with sleeping at one or several homes at their forest farms or rice fields (“tông”). Houses located in the Cambodian villages can either be wooden stilted longhouses inhabited by Jarai extended family or a stilted house occupied by only one nuclear family. Homes located at forest farm plots, and/or on farmers' wet rice fields, are well-constructed stilted bamboo/wooden houses, usually intended for only one nuclear family. The village home is mostly used during the dry season when work on the fields is completed, farmers rest and have their annual ceremonies, celebrations, and planned visits. The rainy season is the most work-intensive period for Jarai farmers, often leading to increased sleeping at forest farms and rice fields. On the Vietnamese side of the border, though the same housing tradition existed originally, residence patterns have changed following the government policy encouraging habitation in modern houses and the incorporation of the Jarai in government-owned plantations. Therefore, most Jarai houses on the Vietnamese border are currently made of concrete without stilts; however, the stilted bamboo/wooden plot huts at farms and fields still exist, and are usually more rudimentary than in Cambodia.

### General sleeping patterns.

#### Cambodia.

About half of Jarai household leaders reported sleeping at forest fields during the malaria transmission season (Table 1), and a majority of (79.3%) reported engaging in deep forest activities (hunting, fishing, and logging), with about one-fourth staying overnight in the forest. Among Jarai youth, sleeping at forest fields during the malaria season was less common (37.0%) while deep forest activities were very common (84.7%) both in boys and girls. However, the proportion of male youths sleeping outside the village during deep forest activities was significantly higher than females (35.8% versus 2.9%; *P* = 0.002) (Table 2).

#### Vietnam.

Reported sleeping arrangements were similar in Vietnam. Deep forest activities and staying overnight in deep forest was less common than in Cambodia among adults and youth (Supplemental Table 1).

### Sleeping outside of the parental home.

Based on the ethnographic study, young Jarai people are expected to become gradually independent and self-sufficient, and therefore they sleep outside their parents' house, spending the night at friends' homes, or in hammocks hung between the stilted houses.

#### Cambodia.

Overall, 40% of the youth was sleeping outside their parents' home (Table 2), and this tended to be more common in boys than girls (51.2% versus 27.5%; *P* = 0.06) (Table 2). Indeed, traditional cultural imperatives indicate that it is less appropriate for girls to sleep outside their parents' homes before marriage. Therefore, among hammock owners, there was a significant difference in hammock use between youth who used them either occasionally (58.5%) or always (35.0%) compared with a majority of male household leaders (83.6%) who almost never used them. However, there was a significant difference in hammock use between boys and girls (*P* = 0.02) since almost all of the former would report using hammocks (occasionally or regularly, 88.6%), while this was the case for only about half of the girls (53.3%). But compared with adults (2.7%), a substantial proportion of male (36.4%) and female (26.7%) youth reported always sleeping in hammocks, and a similar pattern was observed regarding the use of hammock nets (Table 2).

#### Vietnam.

Sleeping outside of the parental home was even more common in Vietnam, for both male (64.0%) and female youth (39.1%). However, differences in hammock use were less pronounced between youth and adults, as less than two-thirds of the youth (63.8%) and more than one-third of household leaders (37.1%) were using them always or sometimes at night. Male youth reported more often than females to always use hammocks at night (31.9% versus 13.6%); however, this difference was not significant (Supplemental Tables 1 and 2).

### Cross-border mobility.

#### Cambodia.

Of the Cambodian Jarai youth, 29.7% regularly spent the night across the border for commercial opportunities such as selling vegetables or for visiting relatives (Table 1). This figure was significantly higher among household leaders (46.3%; *P* = 0.02) corresponding to age-related economic responsibilities. Overall there was no gender difference among youth spending nights across borders (Table 2).

#### Vietnam.

Substantially less border crossings were reported among Vietnamese Jarai youth (15.2%), as well as among the adults (30.0%) compared with Cambodia (Supplemental Table 1).

### Protection with bed nets.

#### Net ownership, use, type, and state.

##### Cambodia.

Although most household leaders reported having a net (95.1%), this number was much lower among youth (55.3%). There was weak statistical evidence that significantly more girls than boys had nets to use (42.6% versus 70.3%; *P* = 0.07; Table 2). Reported ideal net use among net owners was also significantly lower among Jarai youth than among household leaders (84.3% versus 92.7%; Table 1), and again significantly lower among boys than girls (75.4% versus 90.7%; Table 2). The majority of nets being used were untreated nets bought from the local market both among youth (70.6%) and household leaders (58.6%). Moreover, the majority of nets used by youth were torn, while these represented 34% of nets used by household leaders (Table 1). Among youth, significantly more boys than girls used torn nets (61.2% versus 42.7%; Table 2). Youth tended more than adults to perceive that mosquitoes were entering their bed nets (Table 1) and this trend was also seen between boys and girls (Table 2). It was mostly users of intact LLIN or ITN that perceived mosquitoes to be able to enter the net, compared with intact or torn NTN users (data not shown in tables). Qualitative data indicated that the large mesh size of the distributed brand of LLIN influenced the perception that insects could enter despite the insecticide.

##### Vietnam.

Having a bed net among youth was more common in Vietnam (80.3%) than in Cambodia, especially among male youth (73.9%). In contrast to Cambodia, most household leaders (90.4%) and youth (86.5%) reported using ITNs or LLINs that were mostly intact (Supplemental Tables 1 and 2).

#### Categories of protection.

##### Cambodia.

When combining the abovementioned variables following the case definition for protection, only a minority of household leaders and youth slept optimally protected (17.1% and 5.0%, respectively; Table 1), youth being significantly less protected compared with adults. A significantly higher proportion of boys than girls were defined as “unprotected” (72.2% versus 50.0%; Table 2).

##### Vietnam.

Optimal protection was remarkably higher for Vietnamese Jarai household leaders (77.8%) and youth (41.4%), but the difference between youth and adults remained significant as in Cambodia. Similarly, more male youth (53.2%) than female (17.2%) slept unprotected although significance was not reached (Supplemental Table 2).

### High-risk group: young males sleeping outside.

Although Jarai youth generally exhibited lower bed net protection, qualitative data indicated the existence of a high-risk subgroup of male youth sleeping outside parental homes. Young men are given the least priority in the household when designating who requires net protection. Infants and small children along with their mothers have first priority to use nets, followed by adolescent girls, and last the parents and/or adolescent boys. Older children will tend to sleep on separate beds or mats elsewhere in the household, and when space and/or nets become scarce, Jarai youth, particularly males, are expected to seek sleeping arrangements elsewhere, which frequently translates into them sleeping in nonpermanent non-bed spaces often located outside the parental home. In addition, cultural sleeping arrangements define who can share the same bed: while two sisters and also an older sister with her younger, prepubescent brother can share a bed net, older brothers are not allowed to share the same sleeping space with sisters the same age or younger. As a result, the adolescent boy is one of the first to sleep unprotected when there are not enough bed nets available.

In Cambodia, compared with the other male Jarai youth, male “outside sleepers” owned significantly fewer bed nets that were more often torn and stayed overnight in the deep forest and at forest fields more often. As this group required materials for sleeping at night outside the house, they owned significantly more hammocks for individual use (66.3%) compared with other male youths (41.8%; Table 3). Although in Vietnam these differences were less apparent and nonsignificant (except for hammock use at night), there is still a considerable difference in the levels of protection that characterize male outside sleepers and male non-outside sleepers (Supplemental Table 3).

## Discussion

With growing interest in malaria pre-elimination contexts, there is a need to identify and effectively target “hot spots” and similarly “hot populations.”^5^,^10^,^11^ Along the Vietnam–Cambodia border, factors unique to the Jarai youth, males in particular, such as age-specific sleeping patterns and structures, low uptake of preventive measures, and cross-border mobility, increases this subgroup's exposure to malaria. This lack of protection among the young men is due to a combination of factors. They often sleep in nonpermanent sleeping spaces inside and outside the home and, when spending the night in their household, they are given least priority to use the available bed nets, leading to an increased vulnerability to malaria. Furthermore, when sleeping outside their parents' houses, young men are not expected to take a bed net from their parents' house while, at the host family, young visitors are not often granted a bed net.

What explains this behavior in youth more structurally is their status as “youth” and can only be fully understood as embedded in the sociocultural structures that define youth among the Jarai. Because of the transitional character of their social youth status—no longer considered children but not yet adults—fixed and long-term sleeping arrangements are not usually foreseen.^42^ Their mobility, moreover, increases the “flexibility” (and fleetingness) of their sleeping arrangements and, potentially, the likelihood of sleeping unprotected. The relationship between sleeping in ad hoc sleeping spaces and the lower likelihood of using bed nets and the consequent vulnerability of being exposed to malaria in this age group has also been shown across settings in Africa.^43^–^45^ The Jarai youth's mobility is all the more relevant given the prevalence of the main malaria vector *An*. *dirus* in the study region, which is sylvatic and bites early and outdoors.^36^ As illustrated by the low levels of protection among Jarai youth and the comparatively higher mobility of Jarai male youth in both Vietnam and Cambodia, both countries may benefit from adopting similar alternative strategies to address malaria risk in social and/or cultural subgroups that cross-cut borders. Although promoting bed net use may be suitable for risk groups such as forest farmers sleeping at their farms, and might still have some impact among Jarai youth generally, especially in Cambodia, the male Jarai outside-sleeping subgroup requires supplementary measures. Long-lasting insecticidal hammocks (LLIHs), for example, could serve as an effective tool in providing additional protection^46^–^50^ given the already present high use of hammocks among these groups. LLIHs are practical for individuals who are highly mobile, including, but not limited to, Jarai youth and male outside sleepers, who frequently move between houses within the village and between forest and village homes, who carry out activities requiring overnight stays in the deep forest, and who often cross the border. Furthermore, easily transportable and manageable preventive measures, such as LLIHs, could prove attractive for such groups as they would not necessarily require a significant adaptation of established patterns of behavior.

Our results revealed that the estimated protection from malaria infection by bed nets was not only low among Jarai youth, but in fact also lower than expected among Cambodian Jarai household leaders. This difference could be attributed to the methodology used, as most surveys do not inquire about the kind of nets used and whether the net is still intact, and do not include direct observation of these variables, having to rely solely on self-reported data that are often biased so as to meet public health expectations. Although there is a need to identify risk groups, it is unlikely that this can be achieved by regular surveillance activities (i.e. mobile populations) or standardized surveys alone.

### Limitations of the study.

Although directly observing the state of nets is less biased than self-reported state of net, measuring the size and the amount of holes in nets was outside of the scope of the study. Whenever holes were big enough to be observed by the interviewer at first glance, people themselves stated the nets were torn and mosquitoes entered, and the net was not impregnated, we assumed mosquitoes would indeed be able to enter. The main study limitation was the lack of malariometric data of the identified high-risk subgroups, which did not allow for the epidemiological confirmation that they are indeed more at risk for malaria infection than other subgroups. The strength of the study lies exactly in the increased understanding of this social heterogeneity or the existence of differential risk factors and in the identification of one specific bottleneck for the further reduction of malaria in this border setting. In addition, it shows how this risk is an integrated part of the culturally constructed category of youth and that it cannot be understood nor targeted in isolation from this context.

## Conclusion

Standard malaria control tools and strategies developed and directed at majority populations can have a limited impact in contexts where transmission is chiefly restricted to specific areas and/or vulnerable settings and populations. Moreover, specific subgroups display different kinds of vulnerability to malaria and therefore require different approaches to further reduce malaria. The key to capturing this diversity lies in using mixed methods approaches, which allow for in-depth understanding of different sociocultural contexts in relation to malaria exposure, leading to more effective control strategies by tailoring them to specific subgroups at risk.

## Supplementary Material

Supplemental Tables.

## Figures and Tables

**Table 1:** Youth and household leader surveys in Cambodia

	Youth survey (*N* = 300)	Household survey (*N* = 246)	*P* value
	*n* (%)	*n* (%)	
Mobility patterns			
Sleeps at forest fields during malaria season	111 (37.0)	106 (51.4)	0.23
Sleeps outside parents' house (always or sometimes)	121 (40.3)	NA	NA
Goes often to deep forest	254 (84.7)	195 (79.3)	0.42
Spends night in deep forest (always or sometimes)	62 (20.7)	73 (24.8)	0.13
Spends nights across the border in Vietnam	89 (29.7)	114 (46.3)	0.02
Sleeping materials			
Net ownership			
Owns a bed net	166 (55.3)	234 (95.1)	0.0003
Does not own a bed net	134 (44.7)	11 (4.5)	
Missing	–	1 (0.4)	
Net use among net owners			
Never uses a bed net	3 (1.8)	12 (5.1)	0.04
Sometimes uses a bed net	23 (13.9)	5 (2.1)	
Always uses a bed net	140 (84.3)	217 (92.7)	
Net type among net users			
Uses non-treated net	115 (70.6)	130 (58.6)	0.08
Uses treated net	48 (29.4)	88 (39.6)	
Missing	–	4 (1.8)	
State of net among net users			
Intact (or repaired)	81 (49.7)	135 (60.8)	0.18
Broken (or repaired and broken again)	82 (50.3)	75 (33.8)	
Missing	–	12 (5.4)	
Hammock ownership for personal use			
Owns a hammock	103 (34.3)	110 (44.7)	0.29
Does not own a hammock	197 (65.7)	135 (54.9)	
Missing	–	1 (0.4)	
Hammock use for sleeping at night among hammock owners			
Never	17 (16.5)	92 (83.6)	0.002
Sometimes	50 (48.5)	15 (13.6)	
Always	36 (35.0)	3 (2.7)	
Has a hammock net to use while sleeping in hammock	27 (26.2)	10 (9.1)	0.01
Perceived protection of net among net owners			
Mosquitos enter the net			
Yes	79 (47.6)	77 (32.9)	0.19
No	97 (52.4)	157 (67.1)	
Categories of protection			
Unprotected	186 (62.0)	60 (24.4)	0.002
Partially protected	99 (33.0)	127 (51.6)	
Optimally protected	15 (5.0)	42 (17.1)	
Missing	–	17 (6.9)	

**Table 2:** Youth survey by gender in Cambodia

	Male (*N* = 162)	Female (*N* = 138)	*P* value
	*n* (%)	*n* (%)	
Mobility patterns			
Sleeps at forest fields during malaria season	65 (40.1)	46 (33.3)	0.44
Sleeps outside parents' house (always or sometimes)	83 (51.2)	38 (27.5)	0.06
Goes often to deep forest	139 (85.8)	115 (83.3)	0.66
Spends night in deep forest (always or sometimes)	58 (35.8)	4 (2.9)	0.002
Spends nights across the border in Vietnam	50 (30.9)	39 (28.3)	0.65
Sleeping materials			
Bed net ownership			
Has a bed net	69 (42.6)	97 (70.3)	0.07
Does not have a bed net	93 (57.4)	41 (29.7)	
Net use among net owners			
Never uses a bed net	2 (2.9)	1 (1.0)	0.006
Sometimes uses a bed net	15 (21.7)	8 (8.2)	
Always uses a bed net	52 (75.4)	88 (90.7)	
Net type among net users			
Uses non-treated net	39 (58.2)	76 (79.2)	0.006
Uses treated net	28 (41.8)	20 (20.8)	
State of net among net users			
Intact (or repaired)	26 (38.8)	55 (57.3)	0.03
Broken (or repaired and broken again)	41 (61.2)	41 (42.7)	
Hammock ownership			
Has a hammock	88 (54.3)	15 (10.9)	0.009
Does not have a hammock	74 (45.7)	123 (89.1)	
Hammock use for sleeping at night among hammock owners			
Never uses hammock	10 (11.4)	7 (46.7)	0.02
Sometimes uses hammock	46 (52.3)	4 (26.7)	
Always uses hammock	32 (36.4)	4 (26.7)	
Has a hammock net to use among hammock owners	23 (26.1)	4 (26.7)	–
Perceived protection of net among net owners			
Mosquitos enter the net			
Yes	39 (56.5)	40 (41.2)	0.06
No	30 (43.5)	57 (58.8)	
Categories of protection			
Unprotected	117 (72.2)	69 (50.0)	0.05
Partially protected	36 (22.2)	63 (45.7)	
Optimally protected	9 (5.6)	6 (4.3)	

**Table 3:** Youth survey by males sleeping or not sleeping outside their parental home in Cambodia

	Male non-outside sleepers (*N* = 79)	Male outside sleepers (*N* = 83)	*P* value
	*n* (%)	*n* (%)	
Mobility patterns			
Sleeps at forest fields during malaria season	23 (29.1)	42 (50.6)	0.01
Goes often to deep forest	62 (78.5)	77 (92.8)	0.06
Spends nights in deep forest	12 (15.2)	46 (55.4)	0.001
Spends nights across the border in Vietnam	31 (37.3)	19 (24.1)	0.06
Sleeping materials			
Net ownership			
Has a bed net	42 (53.2)	27 (32.5)	0.06
Does not have a bed net	37 (46.8)	56 (67.5)	
Net use among net owners			
Never uses a bed net	2 (4.8)	0	0.42
Sometimes uses a bed net	6 (14.3)	9 (33.3)	
Always uses a bed net	34 (81.0)	18 (66.7)	
Net type among net users			
Uses non-treated net	22 (55.0)	17 (63.0)	0.40
Uses treated net	18 (45.0)	10 (37.0)	
State of net among net users			
Intact (or repaired)	20 (50.0)	6 (22.2)	0.01
Broken (or repaired and broken again)	20 (50.0)	21 (77.8)	
Hammock ownership			
Has a hammock	33 (41.8)	55 (66.3)	0.05
Does not have a hammock	46 (58.2)	28 (33.7)	
Hammock use for sleeping at night			
Never uses hammock	4 (12.1)	6 (10.9)	0.15
Sometimes uses hammock	12 (36.4)	34 (61.8)	
Always uses hammock	17 (51.5)	15 (27.3)	
Has a hammock net to use among hammock owners	10 (30.3)	13 (23.6)	0.59
Perceived protection of net among net owners			
Mosquitos enter the net			
Yes	18 (22.8)	21 (25.3)	0.001
No	24 (30.4)	6 (7.2)	
Categories of protection			
Unprotected	49 (62.0)	68 (81.9)	0.09
Partially protected	22 (27.8)	14 (16.9)	
Optimally protected	8 (10.1)	1 (1.2)	
