# Supplementary material for: High Mobility and Low Use of Malaria Preventive Measures among the Jarai Male Youth along the Cambodia–Vietnam Border
Source: Am J Trop Med Hyg. 2015 Oct 7;93(4):810–8. doi: 10.4269/ajtmh.15-0259 (PMC4596604; doi:10.4269/ajtmh.15-0259)
Supplement: Supplementary file 1 [file SD7.pdf]

Supplemental Table 1
Youth and household leader surveys in Vietnam

	Youth survey (*N* = 198)	Household survey (*N* = 203)	
	n (%)	*n* (%)	*P* value
Mobility patterns			
Sleeps at forest fields during malaria season	73 (36.9)	125 (61.6)	0.08
Sleeps outside parents' house (always or sometimes)	105 (53.0)	NA	NA
Goes often to deep forest	121 (61.1)	106 (52.2)	0.54
Spends night in deep forest (always or sometimes)	21 (10.6)	21 (10.3)	0.61
Spends nights across the border in Cambodia	30 (15.2)	61 (30.0)	0.42
Sleeping materials			
Net ownership			
Has a bed net	159 (80.3)	203 (100.0)	0.07
Does not have a bed net	39 (19.7)	–	
Net use among net owners			
Never	33 (20.8)	6 (3.0)	0.07
Sometimes	13 (8.2)	5 (2.5)	
Always	113 (71.1)	192 (94.6)	
Net type among net users			
Uses non-treated net	17 (13.5)	12 (6.1)	0.24
Uses treated net	109 (86.5)	178 (90.4)	
Missing	–	7 (3.6)	
State of net among net users			
Intact (or repaired)	107 (84.9)	177 (89.8)	0.18
Broken (or repaired and broken again)	19 (15.1)	20 (10.2)	
Has hammock for personal use			
Has a hammock	69 (34.8)	54 (26.6)	0.75
Does not have a hammock	128 (64.6)	149 (73.4)	
Missing	1 (0.5)	–	
Hammock use for sleeping at night among hammock owners			
Never	25 (36.2)	34 (63.0)	0.19
Sometimes	26 (37.7)	17 (31.5)	
Always	18 (26.1)	3 (5.6)	
Has a hammock net to use while sleeping in hammock	9 (13.0)	2 (3.7)	0.75
Perceived protection of net among net owners			
Mosquitos enter the net			
Yes	73 (45.9)	112 (55.2)	0.54
No	86 (54.1)	91 (44.8)	
Categories of protection			
Unprotected	74 (37.4)	6 (3.0)	0.03
Partially protected	42 (21.2)	32 (15.8)	
Optimally protected	82 (41.4)	158 (77.8)	
Missing	–	7	

NA = not applicable.

Supplemental Table 2

Youth survey by gender in Vietnam

	Male (*N* = 111)	Female (*N* = 87)	
	n (%)	*n* (%)	*P* value
Mobility patterns			
Sleeps at forest fields during malaria season	52 (46.8)	21 (24.1)	0.03
Sleeps outside parents' house (always or sometimes)	71 (64.0)	34 (39.1)	0.09
Goes often to deep forest	75 (67.6)	46 (52.9)	0.28
Spends night in deep forest (always or sometimes)	18 (16.2)	3 (3.4)	0.25
Spends nights across the border in Cambodia	16 (14.4)	14 (16.1)	0.52
Sleeping materials			
Bed net ownership			
Has a bed net	82 (73.9)	77 (88.5)	0.32
Does not have a bed net	29 (26.1)	10 (11.5)	
Missing	–	–	
Net use among net owners			
Never	29 (35.4)	4 (5.2)	0.006
Sometimes	9 (11.0)	4 (5.2)	
Always	44 (53.7)	69 (89.6)	
Net type among net users			
Uses non-treated net	4 (7.5)	13 (17.8)	0.26
Uses treated net	49 (92.5)	60 (82.2)	
State of net among net users			
Intact (or repaired)	45 (84.9)	62 (84.9)	0.99
Broken (or repaired and broken again)	8 (15.1)	11 (15.1)	
Hammock ownership			
Has a hammock	47 (42.3)	22 (25.3)	0.12
Does not have a hammock	64 (57.7)	64 (73.6)	
Missing	–	1 (1.1)	
Hammock use for sleeping at night among hammock owners			
Never	12 (25.5)	13 (59.1)	0.13
Sometimes	20 (42.6)	6 (27.3)	
Always	15 (31.9)	3 (13.6)	
Has a hammock net to use among hammock owners	6 (12.8)	3 (13.6)	0.28
Perceived protection of net among net owners			
Mosquitos enter the net			
Yes	36 (43.9)	37 (42.5)	0.78
No	46 (56.1)	40 (46.0)	
Categories of protection			
Unprotected	59 (53.2)	15 (17.2)	0.15
Partially protected	17 (15.3)	25 (28.7)	
Optimally protected	35 (31.5)	47 (54.0)	

Supplemental Table 3

Youth survey by males sleeping or not sleeping outside their parental home in Vietnam

	Male non-outside sleepers (N = 40)	Male outside sleepers (N = 71)	P value
Mobility patterns			
Sleeps at forest fields during malaria season	16 (40.0)	36 (50.7)	0.13
Goes often to deep forest	26 (65.0)	49 (69.0)	0.55
Spends nights in deep forest	4 (10.0)	14 (19.7)	0.43
Spends nights across the border in Cambodia	7 (17.5)	9 (12.7)	0.70
Sleeping materials			
Net ownership			
Has a bed net	32 (80.0)	50 (70.4)	0.18
Does not have a bed net	8 (20.0)	21 (29.6)	
Net use among net owners			
Never	10 (31.3)	19 (38.0)	0.11
Sometimes	2 (6.3)	7 (14.0)	
Always	20 (62.5)	24 (48.0)	
Net type among net users			
Uses non-treated net	1 (4.5)	3 (9.7)	0.65
Uses treated net	21 (95.5)	28 (90.3)	
State of net among net users			
Intact (or repaired)	18 (81.8)	27 (87.1)	0.46
Broken (or repaired and broken again)	4 (18.2)	4 (12.9)	
Hammock ownership			
Has a hammock	15 (37.5)	32 (45.1)	0.30
Does not have a hammock	25 (62.5)	39 (54.9)	
Hammock use for sleeping at night			
Never	5 (33.3)	7 (21.9)	0.02
Sometimes	3 (20.0)	17 (53.1)	
Always	7 (46.7)	8 (25.0)	
Has a hammock net to use among hammock owners	2 (13.3)	4 (12.5)	0.71
Perceived protection of net among net owners			
Mosquitos enter the net			
Yes	17 (42.5)	19 (26.8)	0.35
No	15 (37.5)	31 (43.7)	
Missing	8 (20.0)	21 (29.6)	
Categories of protection			
Unprotected	18 (45.0)	41 (57.7)	0.34
Partially protected	6 (15.0)	11 (15.5)	
Optimally protected	16 (40.0)	19 (26.8)	
